# Lynozyfic (Linvoseltamab): A First‐in‐Class Off‐the‐Shelf T‐Cell Redirector for Refractory Multiple Myeloma

**DOI:** 10.1002/jha2.70182

**Published:** 2025-11-20

**Authors:** Raza Ur Rehman

**Affiliations:** ^1^ Shaikh Khalifa Bin Zayed Al‐Nahyan Medical and Dental College Lahore Punjab Pakistan

1

Letter to the Editor

On July 2, 2025, the FDA approved Lynozyfic (linvoseltamab‐gcpt) for the treatment of relapsed or refractory multiple myeloma (RRMM) in patients who have received at least four prior lines of therapy, including a proteasome inhibitor (PI), immunomodulatory agent (IMiD), and an anti‐CD38 monoclonal antibody. Lynozyfic is administered intravenously and represents the first bispecific antibody targeting both BCMA and CD3 to be approved in this setting. Unlike autologous CAR‐T cell therapy, linvoseltamab requires no lymphodepletion or personalized manufacturing, offering an off‐the‐shelf immunotherapeutic option that engages the patient's T cells in real‐time to eliminate malignant plasma cells.

Multiple myeloma (MM) is a malignant neoplasm of plasma cells characterized by clonal proliferation within the bone marrow and the production of monoclonal immunoglobulins (M‐protein). This leads to organ dysfunction involving bone destruction, renal impairment, anemia, and immunosuppression. MM typically evolves from precursor conditions such as monoclonal gammopathy of undetermined significance (MGUS) and smoldering multiple myeloma (SMM), progressing through a multistep transformation that is driven by accumulating genetic mutations and interactions with the bone marrow microenvironment [1]. Globally, MM accounts for approximately 1% of all cancers and around 10% of hematologic malignancies. It is predominantly a disease of the elderly, with a median age at diagnosis of around 69 years [2]. Standard induction for newly diagnosed patients includes a triplet regimen like bortezomib + lenalidomide + dexamethasone (VRd), followed by autologous stem cell transplantation (ASCT) and maintenance lenalidomide [3]. However, the updated EHA–EMN 2024 Guidelines now recommend quadruplet‐based induction as the preferred frontline approach for transplant‐eligible patients. These include anti‐CD38 monoclonal antibody–containing regimens such as daratumumab + VRd (Dara‐VRd) or isatuximab + VRd (Isa‐VRd), both of which have demonstrated higher rates of measurable residual disease (MRD) negativity and longer progression‐free survival compared with triplet regimens. For transplant‐ineligible or frail patients, the guidelines endorse daratumumab + lenalidomide + dexamethasone (Dara‐Rd) or daratumumab + bortezomib + melphalan + prednisone (Dara‐VMP) as frontline standards due to their favorable tolerability and sustained responses [4]. In the relapsed or refractory setting, the EHA–EMN 2024 consensus expands treatment options to include early use of BCMA‐directed cellular and immune therapies. CAR‐T cell products such as ciltacabtagene autoleucel (cilta‐cel) and idecabtagene vicleucel (ide‐cel) are now considered appropriate from first relapse, especially in patients with high‐risk cytogenetics or relapse within 18 months of initial therapy. In addition, off‐the‐shelf bispecific antibodies such as teclistamab, elranatamab, and linvoseltamab can be incorporated earlier in the disease course, either as bridging or second‐line therapies, offering rapid accessibility without the logistical constraints of autologous CAR‐T manufacturing [4]. Elderly/frail patients typically receive lenalidomide + dexamethasone (Rd) or bortezomib‐based combinations [5]. In triple‐class refractory MM, options like selinexor and belantamab mafodotin offer limited benefit, with short‐lived responses and notable toxicity. While CAR‐T therapies such as ide‐cel and cilta‐cel provide deep remissions, their use is restricted by manufacturing delays and the need for lymphodepletion [6]. Given the challenges posed by CAR‐T therapy logistics and the limited efficacy of existing agents in triple‐class refractory disease, there is a pressing need for a therapy that combines potent T‐cell redirection with immediate availability. Linvoseltamab fulfills this by offering an off‐the‐shelf, bispecific approach that delivers deep, durable responses without the need for lymphodepletion or individualized manufacturing.

Linvoseltamab is a bispecific T‐cell engager (BiTE) that simultaneously binds B‐cell maturation antigen (BCMA) on MM cells and CD3 on T cells. This dual engagement brings cytotoxic T cells into close proximity with myeloma cells, triggering T‐cell activation, immune synapse formation, and targeted killing of the malignant plasma cells. Unlike CAR‐T therapy, linvoseltamab requires no ex vivo cell manipulation, offering a potent, off‐the‐shelf immunotherapeutic strategy capable of inducing deep responses even in heavily pretreated, triple‐class refractory MM patients [7]. In a pivotal phase I/II trial published in Journal of Clinical Oncology, linvoseltamab (200 mg) demonstrated a confirmed overall response rate (ORR) of 64.5%, with 47.3% of patients achieving at least a very good partial response (≥ VGPR) and 23.6% reaching complete response or stringent complete response (CR/sCR). Notably, the median duration of response (DOR) was 29.4 months, reflecting a durable benefit in this heavily pretreated population. Responses occurred rapidly, with a median time to response of 1.2 months. Linvoseltamab, a bispecific antibody targeting BCMA on myeloma cells and CD3 on T cells, triggers potent T‐cell–mediated cytotoxicity without the need for lymphodepletion or individualized cell manufacturing. The safety profile was manageable, with cytokine release syndrome (CRS) limited to early cycles and predominantly Grade 1–2. These results establish linvoseltamab as a highly effective, off‐the‐shelf T‐cell engager for patients with triple‐class refractory MM. Figure 1 presents ORR across prespecified clinical subgroups in patients treated with linvoseltamab 200 mg, showing consistent efficacy across age, race, disease stage, and refractory status [8].

**FIGURE 1:**
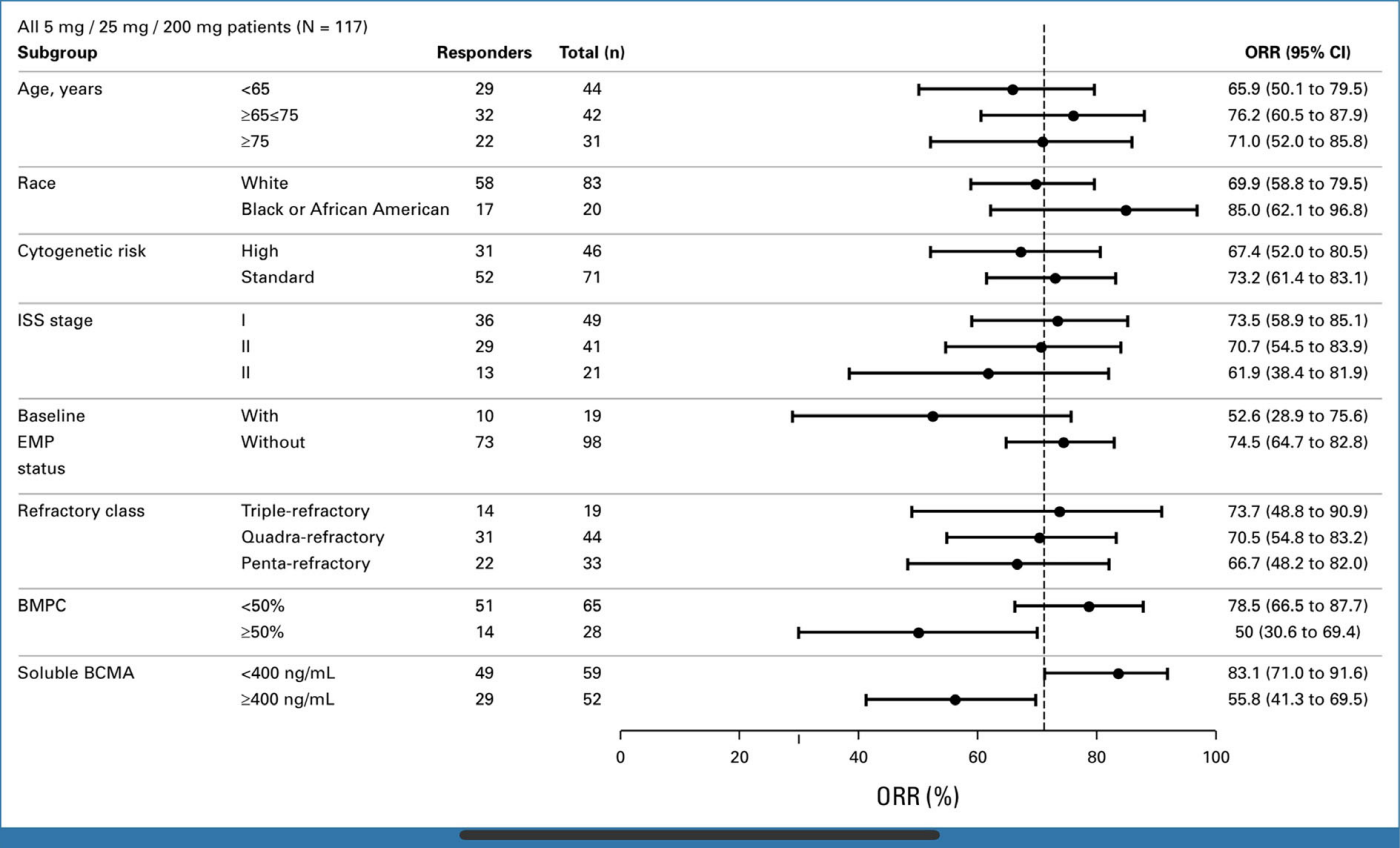
Forest plot illustrating the response rate in prespecified subgroups among 200 mg treated patients. A solid circle denotes the response rate, as determined by the IRC in prespecified subgroups, and whiskers indicate the 95% CI. A vertical dashed line denotes the ORR. High‐risk cytogenetics, presence of del(17p) and/or t(4;14) or t(14;16). BCMA, B‐cell maturation antigen; BMPC, bone marrow plasma cells; EMP, extramedullary plasmacytoma; IRC, independent review committee; ISS, International Staging System; ORR, overall response rate.

Linvoseltamab has demonstrated a favorable and manageable safety profile across early‐phase studies and emerging comparative analyses. In the pivotal Phase I/II trial, CRS occurred in 48.6% of patients, with all cases limited to Grade 1–2 and primarily confined to the initial dosing cycles [8]. Kaplon et al. (2024) highlighted linvoseltamab's lower neurotoxicity incidence compared to other BCMA × CD3 bispecifics, while also noting the benefit of its off‐the‐shelf format that eliminates the need for lymphodepletion [9]. Similarly, Reynolds et al. (2023) reported a lower rate of Grade ≥ 3 infections with linvoseltamab versus teclistamab or talquetamab, attributing this to reduced immunosuppression and the absence of bridging therapy [10]. Collectively, these data suggest that linvoseltamab balances potent T‐cell redirection with a low incidence of serious adverse events, offering a safer immunotherapeutic option for patients with heavily pretreated MM.

## Author Contribution

**Raza Ur Rehman**: conceived the study, conducted the literature review, performed data analysis and interpretation, wrote the manuscript, critically revised the manuscript, approved the final version to be published, supervised the study, and provided administrative support.

## Funding

The author has nothing to report.

## Ethics Statement

Ethical approval was not required for this Editorial.

## Consent

Consent was not required for this editorial as it does not include individual patient data or identifiable information.

## Conflicts of Interest

The author declares no conflicts of interest.

## Data Availability

Data sharing is not applicable to this editorial as no new data were created or analyzed in this study.
